# Embodying Stressful Events: No Difference in Subjective Arousal and Neural Correlates Related to Immersion, Interoception, and Embodied Mentalization

**DOI:** 10.3389/fnbeh.2021.640482

**Published:** 2021-05-13

**Authors:** Sarah De Coninck, Bart Aben, Eva Van den Bussche, Peter Mariën, Frank Van Overwalle

**Affiliations:** ^1^Faculty of Psychology and Educational Sciences, Brain, Body and Cognition and Center of Neuroscience, Vrije Universiteit Brussel, Brussels, Belgium; ^2^Research Units Sustainable Resources, Smart Organizations and Inclusive Society, University College Leuven Limburg, Diepenbeek, Belgium; ^3^Faculty of Psychology and Educational Sciences, Brain and Cognition, KU Leuven, Leuven, Belgium; ^4^Department of Experimental Psychology, Ghent University, Ghent, Belgium; ^5^Faculty of Language and Literature, Centre for Linguistics, Vrije Universiteit Brussel, Brussels, Belgium

**Keywords:** embodiment, interoception, mentalization, immersion, self-referential repetitive thought

## Abstract

Repetitive thought about oneself, including one’s emotions, can lead to both adaptive and maladaptive effects. Construal level of repetitive self-referential thought might moderate this. During *interoception*, which engages areas such as the insula, the anterior and/or posterior cingulate cortex (PCC) and the somatosensory cortex, concrete low level construal self-referential thought is applied, which has been shown to lead to more positive emotions after upsetting events. Contrarily, during *immersion*, related to neural activity in the default mode network (DMN), abstract high level construal self-referential thought is applied, which is linked to depression. The current study investigated whether the integration of concrete and abstract self-referential thought by means of *embodied mentalization* leads to less subjective arousal, decreased DMN activity and increased somatosensory activity as compared to immersion, and to more DMN activity as compared to interoception. In the fMRI scanner, participants imagined stressful events while adopting immersion, interoception or embodied mentalization. After each imagined stressful event, participants rated their subjective arousal and how difficult it was to apply the mode of self-referential thought. Results showed that participants felt that immersion was easier to apply than embodied mentalization. However, no differences in subjective arousal or neural activity were found between immersion, interoception and embodied mentalization. Possible reasons for this lack of significant differences are discussed.

## Introduction

Self-referential repetitive thought is the prolonged, recurrent and repetitive thought about oneself, including one’s emotions (Harvey et al., 2004). It serves as an umbrella term including processes such as rumination, emotional processing, mentalization, immersion, reflection, and interoception (e.g., Martin and Tesser, 1996; Mor et al., 2002; Papageorgiou and Wells, 2003; Watkins et al., 2008; Lebois et al., 2015). An association between self-focus and the likelihood, severity and duration of depression is often found (e.g., Ingram, 1990; Just and Alloy, 1997; Nolen-Hoeksema, 2000; Spasojević and Alloy, 2001). It has been hypothesized that *immersion* or the process of engaging the self in a stressful situation leads to the feeling that a stressful event is subjectively real (Lebois et al., 2015). Lebois et al. (2015) propose that once a subject experiences a negative emotional event as subjectively real negative emotion, physiological stress and rumination will follow.

However, the effects of self-referential repetitive thought are inconsistent (for an overview, see Watkins, 2008). It has been related to poor recovery from emotional events but also to being a necessary component for recovery (Watkins, 2008). One factor moderating whether self-referential thought is adaptive or maladaptive, is whether its content is concrete or abstract (Watkins, 2008). Self-referential thought can comprise high level abstract construals, namely decontextualized mental representations barely entailing the essential gist of events. Self-referential thoughts can also entail low level concrete construals, namely more contextual and specific mental representations containing details of events.

Concrete self-referential thought has more adaptive effects compared to abstract self-referential thought. Among other things, concrete self-referential thought leads to more positive emotions after upsetting events (Rivkin and Taylor, 1999; Watkins, 2004; Moberly and Watkins, 2006), and both more specific autobiographical memory (Watkins and Teasdale, 2001, 2004) and less negative self-judgments in depressed participants (Rimes and Watkins, 2005). Thus it seems beneficial to stimulate and maintain concrete self-referential thought. One way to achieve this is by *interoception* or maintaining an accepting and open awareness of current bodily experiences, even when these experiences are considered negative (Craig, 2003; Hayes, 2004; Lau et al., 2006; Shapiro et al., 2006). This is similar to certain mindfulness interventions (e.g., Farb et al., 2007; Herwig et al., 2010; Lutz et al., 2013; Haase et al., 2014; Fox et al., 2016). On a neurobiological level, Damasio et al. (2000) have shown that neural structures involved in the representation and/or regulation of the bodily state are activated when recalling events representative of a range of emotions. They propose that information about the bodily state processed in the insula, the secondary somatosensory cortex and the anterior and posterior cingulate cortex (PCC) are accessible to consciousness. The insula (e.g., Farb et al., 2007; Herwig et al., 2010; Lutz et al., 2013; Haase et al., 2014; Fox et al., 2016), and the cingulate cortex (e.g., Farb et al., 2007; Herwig et al., 2010; Lutz et al., 2013; Haase et al., 2014; Fox et al., 2016) have repeatedly been implicated when attention is directed toward bodily sensations and emotions. Additionally, several studies found deactivation in the amygdala related to focusing attention toward bodily sensations (Farb et al., 2007; Herwig et al., 2010; Lutz et al., 2013), possibly suggesting reduced emotional arousal.

In contrast, abstract self-referential thought is assumed to be maladaptive. It could be argued that immersion (e.g., Papies et al., 2011, 2014; Tincher et al., 2016) reflects abstract self-referential thought. Even though immersion leads to a vivid experience of sensory detail (Lebois et al., 2015), it is unlikely that this entails a direct experiential awareness of sensations in the current moment, as would be the case for concrete self-referential thought (Teasdale, 1999; Watkins, 2004). Moreover, concrete self-referential thought is expected to be non-evaluative and intuitive whereas abstract self-referential thought is expected to be evaluative and analytical (Teasdale, 1999; Watkins, 2004). The predicted result of immersion is rumination (Lebois et al., 2015), which is typically evaluative and analytical such as abstract self-focused thought (e.g., Watkins and Teasdale, 2001; Raes et al., 2008; Watkins et al., 2008). On a neurobiological level, Fletcher et al. (2010) suggest that processing experiences by means of high level abstract construals relates to activity in the default mode network (DMN; Buckner et al., 2008). DMN activity has been related to internally focused behavior such as self-referential thought (Northoff et al., 2006; Buckner et al., 2008; Qin and Northoff, 2011). Activity in the ventral mPFC (vmPFC), a key region of the DMN (Gusnard et al., 2001; Fox et al., 2005; Qin and Northoff, 2011), has been found during immersion, possibly relating to the engagement of the self in imagined negative events (Lebois et al., 2015).

Despite the maladaptive effects of abstract self-referential thoughts, there are some potential benefits. Abstract high level construals are needed to make inferences and transfer understanding across situations (Vallacher and Wegner, 1987; Förster and Higgins, 2005). They can lead to increased consistency of behavior, especially when working toward long-term goals (Vallacher and Wegner, 1987), and to greater self-control on experimental tasks (Fujita et al., 2006). Given the benefits of both concrete and abstract self-referential thought, the question rises whether it is beneficial to combine both. Luyten et al. (2012) introduced *embodied mentalization*, defined as “the capacity to see the body as the seat of emotions, wishes and feelings and the capacity to reflect on one’s own bodily experiences and sensations and their relationships to intentional mental states in the self and others” (p. 125). The focus on bodily experiences and sensations, relates to concrete self-repetitive thought. However, the focus on processing emotions, wishes, feelings, and intentional mental states in self and others, reflects abstract self-referential thoughts. As such, embodied mentalization integrates both concrete and abstract self-referential thought.

From a neurobiological viewpoint, a network of regions of the DMN consisting of the PCC and the temporoparietal junction, together with the hippocampus might be responsible for an adaptive change in the perspective on the self (Hölzel et al., 2011b). These structures have been shown to increase in gray matter after a mindfulness intervention including interoception (Hölzel et al., 2011a). This leads to the question whether the combination of concrete and abstract self-referential thought employed in embodied mentalization will lead to a more adaptive form of self-referential thought, for example related to activity in PCC and hippocampus, and less negative emotions as compared to immersion.

The goal of the present study is to investigate whether embodied mentalization, an integration of abstract and concrete self-referential thought, could be an adaptive mode of processing emotional events by comparing it to immersion, a mode of abstract self-referential thought, and interoception, a mode of concrete self-referential thought.

First, we will investigate the difference in subjective arousal when applying these different modes of self-referential thought to imagined stressful events. We expect that both interoception and embodied mentalization will lead to less arousal than immersion. Second, we will explore the neural correlates. During interoception, we expect to find activation in the insula, the anterior and/or PCC and possibly the somatosensory cortex (e.g., Damasio et al., 2000; Farb et al., 2007; Herwig et al., 2010; Lutz et al., 2013; Haase et al., 2014; Fox et al., 2016) and deactivation in the vmPFC (Lebois et al., 2015) as compared to immersion. During embodied mentalization, we expect to find activity in the DMN, especially the PCC (e.g., Fletcher et al., 2010; Hölzel et al., 2011a), and the hippocampus (Hölzel et al., 2011b) as compared to interoception, and more activity in the insula as compared to immersion. We also expect to find a decrease in amygdala activation during both interoception and embodied mentalization as compared to immersion (Farb et al., 2007; Herwig et al., 2010; Lutz et al., 2013).

## Materials and Methods

### Participants

Exclusion criteria were a known psychiatric background and using drugs or prescribed medication that could impact attentional or emotion processes. Participants were recruited through flyers distributed at university campuses in the cities of Brussels and Ghent. Thirty participants completed the study. One participant was excluded due to hardware malfunction. The remaining 29 participants (nine men, 20 women) were between 18 and 26 years old (*M* = 21.79, *SD* = 2.11), right-handed, fluent in Dutch and had no neurological antecedents. All of them were university students. The participants received 20 euro for their participation and a copy of the structural MRI scan. This study was approved by the medical ethical committee of the University Hospital of Ghent (where scanning took place, reference: EC/2014/0693) and the University Hospital of Brussels (reference: B.U.N. 143201421684). All participants gave written informed consent.

### Material

Stimuli were one-sentence scenarios depicting stressful events (e.g., “your dad tells you that he has been diagnosed with cancer”). Stressful scenarios were chosen because they lend themselves well to study self-referential thought. To ensure ecological validity, scenarios were based on a database of stressful events (Almeida et al., 2002) and self-report events from undergraduate research assistants (Lebois et al., 2016). Lebois et al. (2016) previously used and normed these stimuli for stressfulness, self-threat, perseverative thought, expectation violation, efficacy, experience, familiarity, plausibility, valence, arousal and certainty. Based on these norms, the 60 most stressful situations were used for the experimental conditions in the current study (20 for each condition), and in a previous neuroimaging study by Lebois et al., 2015). On a 7-point scale, mean ratings for these scenarios were 5.86 (*SD* = 0.37) for perceived stress, 5.67 (*SD* = 0.50) for arousal and 5.82 (*SD* = 0.53) for negative valence. To promote self-engagement, each sentence referred to the participants as “you” and depicted events relevant to college life. 41 additional sentences were chosen, 20 for the baseline task, 15 for the training condition and 6 for the catch trials.

### Design and Procedure

The design contained three experimental conditions (immersion, interoception, embodied mentalization) in a repeated-measures design. Catch trials and an active baseline task were included in the design. Participants were trained in applying the different conditions and responding to the trials. Next, they went through a scanning session and were asked some questions afterward.

#### Training

**FIGURE 1:**
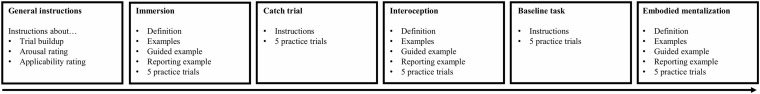
Overview of the training session.

Participants were trained in four conditions, namely a baseline task and three experimental conditions: 1) immersion, 2) interoception, and 3) embodied mentalization. Instructions were based on various therapeutic exercises (for an overview see Gundrum and Stinckens, 2010) and instructions provided in previous research (Papies et al., 2011, 2014; Lebois et al., 2016). Figure 1 gives a schematic overview of the training session.

Participants were told they would have to read and imagine stressful events. They were not told that they had to process emotions, in order to avoid effects of social desirability. Instead, they were told to direct their attention to the events in different ways. It was explained that they would have to provide arousal ratings, referring to a physical feeling of tension independent of how positive or negative they were feeling, and applicability ratings, referring to the degree to which they were able to apply the instructions.

Participants were trained in immersion, interoception, and embodied mentalization in this precise order because these conditions build onto each other (e.g., participants had to understand the interoception instructions before they could learn embodied mentalization). For each condition, the experimenter provided a definition, gave an example and went over three example items with the participant. Afterward, participants practiced five trials on the computer. A shortened version of the instructions for each condition is reported here (see Supplementary Material for a verbatim description):

(1) Immersion: “Absorb yourself in the event as though it is happening at this moment. Try to vividly experience the event in detail.”
(2) Interoception: “Direct your attention toward what you feel in your body, while being aware that the situation is not taking place at this moment. Try to have a friendly and open attention toward your body.”
(3) Embodied mentalization: “Direct your attention toward what you feel in your body and ask yourself “what makes me feel this way?” Stay aware of what is happening in your body when asking yourself this question. Try to be open to everything that arises.”

Participants practiced the catch trials in between training the immersion and the interoception conditions, and the baseline task in between training the interoception and the embodied mentalization conditions.

Before entering the scanner, participants were asked to explain all conditions again in their own words. Based on mentioning important predefined key words for each condition (e.g., for immersion: absorb, put oneself in the event, in detail, as if happening at this moment; also see Supplementary Material), all participants were able to describe the conditions adequately. This method was also used in a similar neuroimaging study by Lebois et al. (2015).

#### Scanning Session

During the fMRI task, participants answered using a response box with four buttons positioned in their left hand.

The task structure was based on a previous neuroimaging study by Lebois et al. (2015). At the beginning of each block the word “break (30 s)” was shown for 30 s. Then a warning was presented, instructing participants to pay attention to the emotion processing strategy instruction for the next block (5 s, “Pay attention! For the next events the task is”), followed by a brief instruction (5 s, e.g., “Direct your attention to your body”).

**FIGURE 2:**
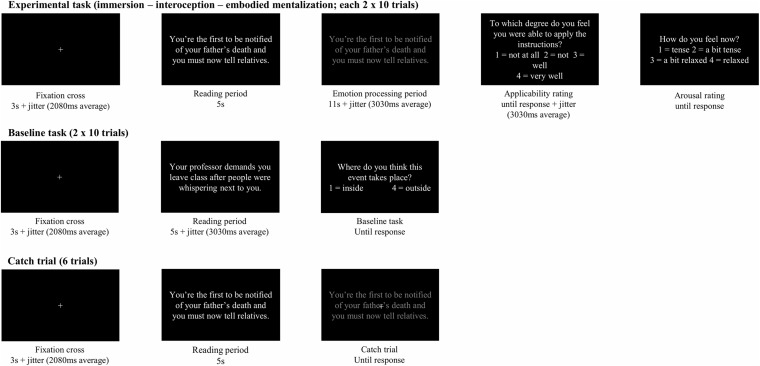
Structure of the trials.

Each trial started with a fixation cross with a duration of 3,000 ms to which a pseudo-logarithmic jitter with an average of 2,080 ms (range: 550–4,400 ms) was added (Hartstra et al., 2010). Next, the sentence describing a stressful event was presented in white font on a black background for 5 s (i.e., reading period), immediately followed by the sentence in gray font for 11 s and a pseudo-logarithmic jitter with an average of 3,030 ms (range 800–6,400 ms Hartstra et al., 2010), indicating that participants had to apply the specific emotion processing strategy (i.e., emotion processing period). Next, they responded to an applicability rating and an arousal rating by means of the response box. Between ratings there was a pseudo-logarithmic jitter with an average of 3,030 ms (range: 800–6,400 ms). The applicability rating asked participants to which degree they felt they were able to apply the emotion processing strategy (1 = not at all, 2 = not, 3 = well, 4 = very well), while the arousal rating asked participants how aroused they felt at that moment (1 = tense, 2 = a bit tense, 3 = a bit relaxed, 4 = relaxed; see Figure 2).

Six catch trials were intermixed with experimental trials at random to ensure that participants would remain focused throughout the whole experiment. A white cross would appear above the gray stimulus indicating that participants had to press a button as fast as possible. A baseline task was presented as a separate block. This baseline task was not included in the analyses.

The four conditions—three experimental conditions and the baseline task—were each presented twice in blocks of 10 trials. Blocks appeared in a pseudo-random order with the premise that each condition appeared once both in the first and second half of the experiment. The order of blocks was counterbalanced between participants. There were three possible orders in which the blocks could appear. The baseline task was always provided in the fourth and eighth block, separating the first and the second half of the experiment. The order of the experimental conditions was organized by means of a Latin square, and was different in the two halves of the experiment.

#### Postscanning

As a final manipulation check, participants were asked to explain how they applied the experimental conditions. Based on mentioning predefined keywords (see Supplementary Material), all participants described the conditions adequately.

#### Imaging Procedure

Images were collected with a Siemens Magnetom Trio TIM scanner system (Siemens Medical Systems, Erlangen, Germany) using a 32-channel radiofrequency head coil. Stimuli were projected onto a screen at the end of the magnet bore that participants viewed by way of a mirror mounted on the head coil. Stimulus presentation was controlled by E-Prime 2.0^1^ (Psychology Software Tools, Pittsburgh, PA) running under Windows XP. First, high-resolution anatomical images were acquired using a T1-weighted 3DMPRAGE sequence [TR = 2,530 ms, TE = 2.58 ms, TI = 1,100 ms, acquisition matrix = 256 × 256 × 176, sagittal FOV = 220 mm, flip angle = 7°, voxel size = 0.9 × 0.86 × 0.86 mm^3^ (resized to 1 × 1 × 1 mm^3^)]. Second, a fieldmap was calculated to correct for inhomogeneities in the magnetic field (Cusack and Papadakis, 2002). Next, whole-brain functional images were collected in a single run using a T2^∗^-weighted gradient echo sequence, sensitive to BOLD contrast (TR = 2000ms, TE = 35ms, image matrix = 64 × 64, FOV = 224 mm, flip angle = 80°, slice thickness = 3.0 mm, distance factor = 17%, voxel size = 3.5 × 3.5 × 4.0 mm^3^, resized to 2 × 2 × 2 mm, 30 axial slices).

#### Image Preprocessing

The fMRI data were preprocessed to remove sources of noise and artifact and analyzed using SPM12 (Wellcome Department of Cognitive Neurology, London, United Kingdom). Functional data were corrected for inhomogeneities in the magnetic field using the fieldmap and for differences in acquisition time between slices for each whole-brain volume, realigned to correct for head movement, and co-registered with each participant’s anatomical data. The functional data were then transformed into a standard anatomical space (2 mm isotropic voxels) based on the ICBM152 brain template (Montreal Neurological Institute). Normalized data were spatially smoothed (6 mm full-width at half-maximum, FWHM) using a Gaussian Kernel. Finally, the preprocessed data were examined for excessive motion artifacts and for correlations between motion and experimental design, and between global mean signal and experimental design, using the Artifact Detection Tool software package (ART)^2^. Outliers were identified in the temporal differences series by assessing between-scan differences (Z-threshold: 3.0 mm, scan to scan movement threshold: 0.5 mm; rotation threshold: 0.02 radians). For each movement outlier (i.e., “bad” scan), a single regressor was included for the analysis. No substantial correlations between motion and experimental design or global signal and experimental design were identified. A default high-pass filter of 128 s was used and serial correlations were accounted for by the default auto-regressive AR(1) model.

### Statistical Analysis

#### Statistical Analysis of fMRI Data

Analyses of the fMRI data at the first (individual) level were conducted using the general linear model of SPM12. For each of the three experimental conditions, five onset regressors were defined (time locked at the beginning of reading, emotion processing, applicability rating, arousal rating, and for the catch trials). Six motion parameters from the realignment as well as all outlier time points (identified by ART) were included as nuisance regressors. The regressors were convolved with a canonical hemodynamic response with event duration set to 0 for all conditions. Six *t*-contrasts were computed for each participant comparing the different conditions to each other during the emotion processing period.

Individual contrast maps were subjected to second-level random effects models. Significance was tested through one-sample *t*-tests. Given that a recent meta-analysis found that acceptance-based strategies were mainly related to deactivations (Messina et al., 2021), both directions of the contrasts between emotion processing strategies during the emotion processing period were looked at (immersion vs. interoception, immersion vs. embodied mentalization and interoception vs. embodied mentalization). No clusters survived an FWE-corrected voxel-level threshold of *p* < 0.05 and a cluster-level threshold of *p* < 0.001 uncorrected. Since no clusters survived this strict threshold, we looked at the more lenient voxel-level threshold of *p* < 0.001 uncorrected and a cluster-level FWE-corrected threshold of *p* < 0.05. Again no clusters survived.

To explore our specific hypotheses, we also conducted *a priori* ROIs centered around MNI coordinates of the insular cortex (−44 10 4; Fox et al., 2016), the dorsal anterior cingulate cortex (−6 18 44; Fox et al., 2016), the PCC (−8 −56 39; Fox et al., 2016), the vmPFC (4 38 −20; Yang et al., 2020), the hippocampus (−26 −28 −17, 26 −33 −15; Spreng et al., 2009) and the amygdala (−23 −3 −21; Kober et al., 2008). For all these ROIs, we constructed spheres with a radius of 10 mm around the center.

#### Statistical Analysis of Subjective Ratings

To see whether there was a difference between conditions in subjective arousal and the difficulty of applying the conditions, repeated measures analysis of variance (ANOVA) were conducted. To increase the power of these analyses, ANOVAs were carried out within stimuli (*n* = 60) instead of within subjects (*n* = 29). Effect sizes for ANOVAs are reported as omega squared (Maxwell and Delaney, 2004). When repeated measures ANOVAs indicated a significant main effect, pairwise comparisons with Bonferroni correction for multiple comparisons were carried out between emotion processing strategies to see which contrasts were significant. Effect sizes for comparisons of means are reported as Cohen’s d calculated with the average standard deviation of both repeated measures as a standardizer (Lakens, 2013).

## Results

### Whole Brain Analyses and *a priori* ROIs

The contrasts of interest and the *a priori* ROIs showed no significant results (see Supplementary Table 1).

### Self-Report Measures

**FIGURE 3:**
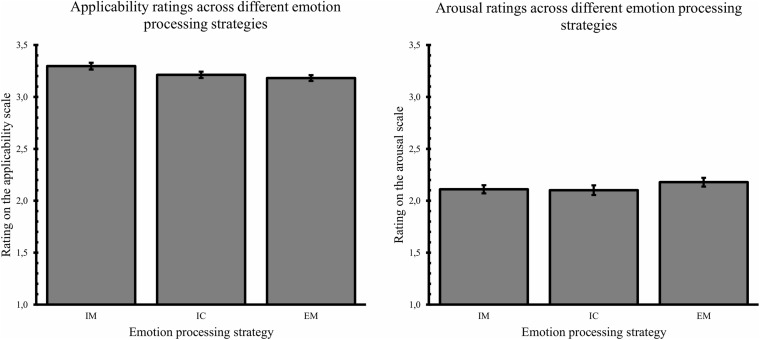
Applicability rating and arousal rating across different emotion processing strategies (IM, immersion; IC, interoception; EM, embodied mentalization). Error bars represent standard errors.

To look at differences in difficulty applying the conditions, a repeated measures ANOVA across stimuli with emotion processing strategy as within-subjects factor and applicability ratings as dependent variable was carried out. This revealed a significant main effect of condition, *F*(2, 118) = 3.53, *p* = 0.03, ω^2^ = 0.03 (see Figure 3). Pairwise comparisons revealed no difference in experienced difficulty between interoception (*M* = 3.18) and embodied mentalization (*M* = 3.16), *d*_*av*_ = 0.10, *p* = 1, while immersion (*M* = 3.27) was rated as less difficult to apply than embodied mentalization (*d*_*av*_ = 0.46, *p* = 0.02) but not interoception (*d*_*av*_ = 0.36, *p* = 0.18; see Figure 3).

The same repeated measures analysis conducted with the arousal ratings as dependent variable, revealed no effect of emotion processing strategy on subjective arousal, *F*(2, 118) = 1.65, *p* = 0.20, ω^2^ = 0.006.

## Discussion

The current study investigated emotional and neural effects of concrete and abstract self-referential thought while processing stressful events. Embodied mentalization (integration of concrete and abstract), interoception (concrete) and immersion (abstract) were compared with respect to their effect on subjective arousal and neural activation in relevant brain areas. Contrary to our hypotheses, no differences in subjective arousal or neural activation were found. Participants did find immersion more easy to apply than embodied mentalization. This might be because individuals spontaneously engage in repetitive thought, projecting themselves in future and past hypothetical situations (Killingsworth and Gilbert, 2010). This seems similar to the immersion instructions which required participants to project themselves in the events as if they were happening at the current moment. Conversely, embodied mentalization required a present moment awareness of bodily sensations integrated with abstract self-referential thought, which might be a less spontaneous strategy. It therefore seems plausible that embodied mentalization was the most novel and was experienced as more difficult than immersion.

The lack of differential neural activation between interoception and immersion is surprising, given that such differences have been found in previous neuroimaging research (e.g., Farb et al., 2007; Lebois et al., 2015). We speculate that learning embodied mentalization, which adds onto interoception, might change the way participants apply interoception, even when they are instructed to only apply interoception. During the training, participants already learn the three different strategies. Because of this, they learn that interoception can be part of a more complex strategy such as embodied mentalization. Somewhat in line with this, some studies find different neural activations related to mindfulness based on the degree of experience with mindfulness (e.g., Taylor et al., 2011). Taylor et al. (2011) have suggested that novices take a more active approach to downregulating emotions, akin to reappraisal, while experts use a more acceptance based approach. This illustrates that the same emotion processing strategy can be applied differently depending on context.

The lack of significant results might be related to some limitations of the current study. First, only a short training session was used to teach participants the different emotion processing strategies. This was done since our aim was to investigate how different emotion processing strategies might present themselves in daily life. However, novices might apply introspective emotion processing strategies differently compared to experienced participants. This is in line with mindfulness research (e.g., Taylor et al., 2011), where focusing on bodily sensations led to a downregulation of the left amygdala in novices (Herwig et al., 2010; Taylor et al., 2011; Lutz et al., 2013), but not in experienced participants (Taylor et al., 2011). Aggregating neuroimaging data across individuals for processes which are highly dependent on individuals’ experience seems problematic (Van Dam et al., 2017). Given that the idea of embodied mentalization is relatively novel and difficult to apply, a more intense training might be needed to disentangle the neurobiological processes related to immersion, interoception and embodied mentalization. On the other hand, if the difference in difficulty applying embodied mentalization obscured our results, we would have expected to see different neural activity in regions related to complexity or cognitive load. Other studies have found activation in parietal regions related to complexity in human reasoning (Kroger et al., 2002) and cognitive load in a working memory task (Arsalidou et al., 2013). Given that no differences in brain activation between immersion and embodied mentalization were found, the difference in difficulty was probably not meaningful enough to have an effect on neural activations^3^. Moreover, even though embodied mentalization was found to be more difficult to apply than immersion, participants still indicated being able to apply embodied mentalization.

Second, there is no straightforward way to check whether participants accurately applied the emotion processing strategies. The current study made use of the judgment of the experimenter to assess whether participants were correctly applying the different strategies (based on predefined keywords). Although this is a method that has previously been used (e.g., Lebois et al., 2015), future research could use a writing task or a verbal report of the internal thoughts of participants and look at the use of certain key elements (such as concreteness). Previous behavioral studies have successfully made use of independent blind judges to differentiate between concrete and abstract self-referential processing (e.g., Watkins et al., 2008; Galfin and Watkins, 2012). However a writing task or verbal report can only be used during the training and not during the scanning session.

A third limitation is that the emotion processing phase started 5 s after the stressful scenario appeared on the screen. It might take some time to attain a process of immersion, interoception or embodied mentalization. A previous neuroimaging study by Lebois et al. (2015) used the timing of 6.9 s for reading and 6.9 s for emotion processing. The current study lengthened the emotion processing strategy period to maximize the time participant could use to attain immersion, interoception or embodied mentalization. In addition, strategies were provided in blocks so participants could obtain one strategy for an entire block. It is impossible to know at which moment participants have adequately reached this mode of processing emotions. Another way of approaching this in the future, is by having participants press a button when they have reached a state of interoception, embodied mentalization or immersion (e.g., Damasio et al., 2000). Nonetheless, this might confound the introspective processes with an evaluative element.

Fourth, the nature of the emotional stimuli might influence the results of the different emotion processing strategies. Imagining stressful situations and applying a newly learned emotion processing strategy might be very taxing. Moreover, during embodied mentalization participants were encouraged to think about the self-relevance of the emotional situation (e.g., “what makes me feel this way”). It seems logical that these strategies might be better suited to emotional situations that are highly self-relevant. In the current study it was not clear how self-relevant the situations were for the participants.

Fifth, it is possible that our study was not sufficiently powered. Given that there are several difficulties with effect sizes in neuroimaging studies (e.g., Reddan et al., 2017), they are not commonly reported in fMRI studies. We therefore were not able to estimate effect sizes beforehand. Our sample size was based on common sample sizes in imaging research (Szucs and Ioannidis, 2020). Cremers et al. (2017) showed that for strong localized effects a sample size of 20 reaches sufficient power, whereas if the true effect is weak and diffuse, even a sample size of 150 still reaches low power. This suggests that our sample size should have been sufficient to detect large localized effects, but not to detect weak diffuse effects. A recent study also pointed out that replicability of neuroimaging results is better for larger sample sizes, especially for localized effects (Bossier et al., 2020).

In conclusion, uncertainty remains around which modalities of self-referential thought are adaptive. Specifically high level construal self-referential thought has often been found to be maladaptive (for an overview, see Watkins, 2008). On the other hand, high level construal self-referential thought plays a crucial role in transferring knowledge across situations and working consistently toward long-term goals (Vallacher and Wegner, 1987; Förster and Higgins, 2005). In the past decade, the importance of low level construal self-referential thought in the form of focusing on bodily sensations in clinical practice has been recognized (e.g., Payne et al., 2015; Price and Hooven, 2018). For example, mindfulness-based therapy is widely recognized as an effective treatment (e.g., Hofmann et al., 2010; Khoury et al., 2013). Röhricht (2009) pointed out that including bodily practices in psychotherapy can be especially valuable for mental disorders with limited treatment response to traditional talking therapy. In order to widely include a focus on bodily sensations into clinical practice, a strong evidence base needs to be developed. Yet little is known about how a low level construal focus on the body can be adaptively integrated with more abstract self-referential thought. Future research can build upon the lessons learned in this study to investigate the integration of low construal and high construal self-referential thought during emotion processing more thoroughly.

## Data Availability Statement

The datasets presented in this article are not readily available because they contain personal data which cannot be shared without restrictions under the GDPR. The data were collected before the current GDPR regulations became operational. As a result, we did not explicitly ask participants’ consent to share their data. Furthermore, we provided participants with their structural scans, which they could have shared through social media. This implies that even efforts to de-identify the data might not ensure that data are fully anonymous (see also White et al., 2020). De-identified data of this kind cannot be shared without the consent of participants under the GDPR. Requests to access the datasets should be directed to SDC.

## Ethics Statement

The studies involving human participants were reviewed and approved by the Medical Ethical Committee of the University Hospital of Ghent and the Medical Ethical Committee of the University Hospital of Brussels. The patients/participants provided their written informed consent to participate in this study. Written informed consent was not obtained from the individual(s) for the publication of any potentially identifiable images or data included in this article.

## Author Contributions

SDC, PM, and FVO contributed to conception and design of the study. SDC and BA acquired the data. SDC and BA performed the statistical analysis. SDC, BA, EVDB, and FVO contributed to the interpretation of the data. SDC wrote the manuscript. All authors contributed to the manuscript revision, read, and approved the submitted version.

## Conflict of Interest

The authors declare that the research was conducted in the absence of any commercial or financial relationships that could be construed as a potential conflict of interest.

## Footnotes

1 www.pstnet.com/eprime

2 http://www.nitrc.org/projects/artifact_detect

3 Based on a suggestion of the reviewers, we looked into spheres with a radius of 10 mm around the center of coordinates found for 3 peaks in a meta-analysis on cognitive difficulty (43, −41 44, −50 8 47, −39 −43 54; Radua et al., 2014). This again yielded no significant results.
