# Supplementary material for: Embodying Stressful Events: No Difference in Subjective Arousal and Neural Correlates Related to Immersion, Interoception, and Embodied Mentalization
Source: Front Behav Neurosci. 2021 May 13;15:640482. doi: 10.3389/fnbeh.2021.640482 (PMC8161507; doi:10.3389/fnbeh.2021.640482)
Supplement: Supplementary file 1 [file Table_1.docx]

Supplementary Material

# Instructions for training the different emotion processing strategies

“In this experiment, you will read about different events. You will be asked to direct your attention in different ways to these events. I will teach you different strategies to do this, so that you will know what to do when you’re in the fMRI scanner.

When you’re in the scanner, there will be moments when you will see an instruction that refers to one of the strategies that you will now learn. After receiving this instruction, you will get to read different events. You should apply the strategy (to which the instruction refers) to these events.

Every event will first appear in white font on the screen. As long as the font is white, you should read the sentence and try to imagine the event. When the font turns grey, you should start to apply the specific strategy.

For every event, you will be asked to answer two questions, to what degree you were able to apply the instructions (referring to the strategies) and how tense you feel. People can mean different things when they are talking about tension. In this experiment tension means a bodily feeling of arousal (e.g. your heart beating faster, your breathing goes faster, your stomach cramps together, …). You should think of tension as being independent of how positive or negative you feel. For example, you can feel tense when you’re going on a rollercoaster, or when you’re in love, but at the same time you’re feeling positive.

In a moment, you will practice all of this on the computer and everything will become more clear.”

1. **Absorb yourself in the event (*Immersion*)**

*Definition (key words: absorb, put oneself in the event, in detail, as if happening at this moment)*

“When you see the instruction ‘Take in the event, try to absorb yourself in the event’ on the screen, we want you to absorb yourself into the event as if it is happening right at this moment. In your mind, try to travel to the event that you will read on the screen, and try to vividly experience every detail of the event. Ask yourself what you would see, hear and feel in this event. Think about what would go through your mind if you would be in the event. Try to see and experience the event in every detail, from moment to moment, like in a film.”

*Examples*

“For example, think about a time when you had to give a presentation and someone asked you a question that you couldn’t answer. Absorb yourself completely in this event and re-experience the event. Go back in time to the moment and place where the experience happened. Now imagine that it is playing out before your eyes. Re-experience everything in vivid detail.

- You see the space filled with people who are all wearing differently colored clothes.
- You hear the question being posed.
- You feel nervous.
- You hear yourself mumbling an answer
- You hear the uncomfortable silence that follows your answer.
- You feel uncomfortable.
- You think about other things you could have said.

When you are absorbing yourself, it seems as if you’re having a vivid daydream that you are really experiencing. By going into detail, it seems to be real, as if it’s happening right now.”

*Example guided by the researcher:*

“We are going to go through an example together.”

*(Read the first example presented on the computer)*

“Read the event and try to immerse yourself in it.”

*(Give the participant some time)*

- “Can you tell me what you are seeing in this event?
- Can you tell me what you are feeling in this event?
- Can you tell me what is going through your mind in this event?”

*(If necessary, ask clarifying questions to let the event unfold more)*

*Examples with reporting to researcher:*

“We are going to read another event on the computer. This time, try to absorb yourself in the event and tell me what you feel, hear and think without me asking guiding questions.”

*(Participants read two events, immerse themselves and report their experiences to the researcher)*

*Examples on the computer*

“I will let you practice some trials on the computer. You will read the instruction in the same way that you will read it when you are in the scanner. After the instruction, you will see the event.

As mentioned, the text of the event will first be white. When the text turns grey, you should begin to absorb yourself in the event. After every event you will be asked some questions. You can answer these questions by pressing the number keys. After these trials, you can come and get me.”

*(Participants go through 5 example trials on the computer)*

1. **Catch trial**

“There will be some trials where we will want to check whether you are paying attention to the task. These trials can happen within every strategy. You will not get an instruction that will announce this kind of trial, as you just did for the ‘absorb yourself’ strategy. When these trials happen, you will read the event, just as with the other trials. Instead of the text turning grey, you will see a white cross appearing. When you see the white cross, you should press ‘1’ as soon as possible. In the scanner this will be the left key. I will let you practice some of these trials. Afterwards, you can come and get me.”

*(Participants go through 5 example trials on the computer)*

1. **Bodily reactions (Interoception)**

*Definition (key words: acceptance, stay in the present, attention towards the body)*

“When you see the instruction ‘direct your attention to your body’ on the screen, we want to ask you to direct your attention to what you feel in your body when you are reading about the event. Contrary to what you did during absorption, try to stay aware that the event is not happening in this moment.

During this strategy, we are asking you to bring your attention through your whole body. Which sensations do you notice? You may not notice any sensations at all. That is completely okay. Everything you may or may not experience is okay. The quality of your attention is friendly and loving. It’s not about feeling something special or relaxing. It’s about being open to everything that arises in your body, even if it is unpleasant.

Every once in a while, you will notice that your attention digresses or that you’re ‘gone’ for a moment. Don’t worry, that is the way our mind works. When this happens, try to friendly direct your attention back to your body.”

*Examples*

“Think about the example I gave you when we were learning the absorption strategy. Think about a presentation you gave in the past were a question was posed that you could not answer. Instead of fully absorbing yourself in the event, pay attention to the bodily sensations that you are experiencing in this moment, when you are reading this event. When doing this, bring your attention through your whole body in an accepting way.

- You bring your attention through your body and explore where you can feel any sensations.
- You notice that your heart is beginning to beat faster.
- You notice your stomach contracting.
- You notice your breathing accelerating.

At the same time, you notice that the event is not taking place at this moment:

- You feel your feet on the ground.
- You feel your behind on the chair.
- You feel your hands on the *cold* (*depending on situation*) table.
- You notice your breathing slowing down again.
- Maybe you don’t feel anything, in this case you try to keep your attention directed at your body and explore whether you might feel anything.”

*Example guided by the researcher*

“We are going to go through an example together.”

*(Read the example on the computer screen)*

“Read the example and try to pay attention to how your body reacts to the event.”

*(Give the participant some time)*

- “Do you notice anything specific in your body when you’re reading this event?
- Bring your attention through your entire body.
- What do you feel in your feet / legs / behind… stomach area… ?”

*(If necessary, ask more clarifying questions)*

*Examples with reporting to the researcher*

“We are going to read another event on the computer screen. Try to bring your attention towards your body and tell me what you’re experiencing without me asking guiding questions.”

*(Participants read two events themselves, pay attention to their body and report to the researcher)*

*Examples on the computer*

“I will let you practice some trials on the computer. The instructions will be presented in the same way as they will be presented later on when you’re in the scanner.

After the instructions, you will see the events. As mentioned, the text of the event will first be white. When the text turns grey, you should begin applying the ‘pay attention to your body’ strategy. After every event you will be asked some questions. You can answer these questions by pressing the number keys. After these trials, you can come and get me.”

*(Participants go through 5 example trials on the computer)*

1. **Baseline**

“There will be another task that you can be asked to do when you’re in the scanner. In this task, you will be asked to judge whether the event is taking place inside or outside. You’ll get the instruction ‘Pay attention to where the event is taking place’ when asked to do this task. There are no right or wrong answers, just try to estimate as best you can where you think the event takes place. I will let you practice some trials on the computer. First you will read the event, as you did in the other tasks. Then you will be asked whether the event takes place inside or outside. You can respond to this with the number keys on the computer, and the left and right keys in the scanner. Afterwards, you can come and get me and we will go over the final strategy.”

*(Participants go through 5 example trials on the computer)*

1. **What makes you feel these bodily reactions (Embodied mentalization)**

*Definition (key words; asking “what makes” in a curious and open way, present, bodily reactions)*

“When u see the instruction ‘What makes you feel these bodily sensation?’ on the screen, we want to ask you to direct your attention towards the bodily sensations that you feel when reading the events. Stay aware of what you’re feeling in your body at this moment and ask yourself ‘what makes me feel this way?’. Take the time to feel what arise in your body after asking yourself this question.

Try to have a friendly open attention towards everything that arises when you ask yourself this question. This can be different things like thoughts, images, feelings, sensations, … Try to be open to everything that arises. So we want you to ask yourself what makes you feel these bodily sensations. Try to stay in contact with everything that arises in your body when you ask yourself this question. Maybe you can feel something shift in your body when you ask yourself this question.”

*Examples*

“Think about the example of the presentation again. When applying the ‘what makes you feel these bodily reactions’ strategy, you remain aware that you are sitting in this chair in this chamber. You notice yourself having bodily reactions when reading about the event. You ask yourself in a curious way what makes you feel this way. You try to stay open to everything that arises.

- You explore what you’re feeling in your body when reading the event
- You notice that your heart starts to beat faster
- You feel your stomach contract
- You ask yourself what makes you feel these bodily sensations
- You notice that you’re feeling nervous or anxious and that this makes your heart pound faster
- You think about how important it is for you to be able to be good at presenting (because you want to do something with it in your career, or there might be someone important in the public)
- You notice that your bodily sensations make you feel a kind of healthy stress that helps you present better
- You see a kind of image of yourself as a caterpillar evolving into a butterfly by putting yourself into these kind of events
- You notice your heart rate slow down when you turn your attention back to your bodily sensations”

*Example guided by the researcher*

“We are going to go through an example together.”

*(Read the example on the computer together)*

“Read the example and try to pay attention to what arises in your body afterwards.”

*(Give the participant some time)*

- “Do you notice anything specific when you are reading this event?
- Describe what you’re experiencing when you ask yourself what makes you feel this way.
- Pay attention to your bodily sensations and notice whether anything has changed.”

*(If necessary, ask more clarifying questions)*

*Examples with reporting to the researcher*

“We are going to read another event on the computer screen. Try to ask yourself what makes you feel your bodily sensations and tell me what you’re experiencing without me asking guiding questions.”

*(Participants read two events themselves, apply ‘what makes you feel these bodily sensations’ and report to the researcher)*

*Examples on the computer*

“I will let you practice some trials on the computer. The instructions will be presented in the same way as they will be presented later on when you’re in the scanner. After the instructions, you will see the events. As mentioned, the text of the event will first be white. When the text turns grey, you should begin applying the ‘what makes you feel your bodily sensations’ strategy. After every event you will be asked some questions. You can answer these questions by pressing the number keys. After these trials, you can come and get me.”

*(Participants go through 5 example trials on the computer)*

1. **Final check to see whether participants understand the different strategies**

“Can you tell me one last time what you should be doing when applying each strategy”?

*(Participants should mention the key words below for every strategy):*

*Immersion: absorb, in detail, as if happening in this moment*

*Interoception: accept, stay in the present, attention towards bodily experiences*

*Embodied mentalization: curious openness towards “what makes me feel”, stay in the present, bodily experiences*

# Table 1: Non-significant results of the whole brain analysis

| Anatomical labels | x | y | z | cluster size | p | t | p |
| --- | --- | --- | --- | --- | --- | --- | --- |
| Immersion > Interoception | | | | | | | |
| R Precuneus | 14 | -46 | 42 | 50 | 0,51 | 5,16 | 0,35 |
| R Superior Frontal Gyrus | 18 | 26 | 48 | 98 | 0,12 | 4,47 | 0,84 |
| R Middle Frontal Gyrus | 32 | 22 | 44 |  |  | 4,16 | 0,97 |
| R Superior Frontal Gyrus | 26 | 26 | 50 |  |  | 3,86 | 1,00 |
| L Middle Frontal Gyrus | -34 | 28 | 42 | 44 | 0,60 | 4,38 | 0,89 |
|  |  |  |  |  |  |  |  |
| Immersion > Embodied mentalization | | | | | | | |
| L Middle Occipital Gyrus | -30 | -80 | 40 | 41 | 0,70 | 4,54 | 0,74 |
|  |  |  |  |  |  |  |  |
| Interoception > Immersion | | | | | | | |
|  | -10 | -40 | 24 | 13 | 0,99 | 4,54 | 0,80 |
|  |  |  |  |  |  |  |  |
| Interoception > Embodied mentalization | | | | | | | |
| No suprathreshold clusters were found | | | | | | | |
|  |  |  |  |  |  |  |  |
| Embodied mentalization > Immersion | | | | | | | |
| R Middle Frontal Gyrus | 50 | 0 | 54 | 10 | 0,99 | 4,58 | 0,71 |
| R Caudate Nucleus | 10 | 12 | -2 | 15 | 0,98 | 4,06 | 0,97 |
| R Rolandic Operculum | 44 | 8 | 14 | 21 | 0,94 | 3,85 | 1,00 |
| R IFG (p. Triangularis) | 40 | 12 | 22 |  |  | 3,50 | 1,00 |
| L Precentral Gyrus | -54 | -2 | 22 | 20 | 0,95 | 3,81 | 1,00 |
| L Precuneus | -8 | -70 | 42 | 17 | 0,97 | 3,73 | 1,00 |
|  |  |  |  |  |  |  |  |
| Embodied mentalization > Interoception | | | | | | | |
|  | -2 | -12 | -6 | 16 | 0,97 | 5,35 | 0,27 |
| R Postcentral Gyrus | 62 | -8 | 18 | 25 | 0,88 | 5,34 | 0,28 |
| L Superior Temporal Gyrus | -46 | -28 | 4 | 26 | 0,86 | 4,86 | 0,60 |
|  | 8 | -24 | -6 | 14 | 0,98 | 4,46 | 0,88 |
| L Heschls Gyrus | -48 | -14 | 6 | 34 | 0,73 | 4,45 | 0,89 |
| R Inferior Temporal Gyrus | 40 | -66 | -6 | 34 | 0,73 | 4,37 | 0,93 |
|  | 36 | -60 | -2 |  |  | 3,90 | 1,00 |
| L Postcentral Gyrus | -58 | -16 | 34 | 43 | 0,57 | 4,29 | 0,95 |
| R Superior Medial Gyrus | 16 | 62 | 8 | 11 | 0,99 | 4,28 | 0,95 |
| R Inferior Temporal Gyrus | 42 | -54 | -12 | 11 | 0,99 | 4,24 | 0,97 |
| R SupraMarginal Gyrus | 64 | -18 | 26 | 22 | 0,92 | 4,08 | 0,99 |
| R Angular Gyrus | 48 | -58 | 40 | 80 | 0,17 | 4,05 | 0,99 |
| R Angular Gyrus | 48 | -60 | 32 |  |  | 3,77 | 1,00 |
|  | 4 | -30 | -10 | 18 | 0,96 | 3,87 | 1,00 |

Note: x, y, and z = Montreal Neurological Institute (MNI) coordinates of the peak values; t = t-score of the peak values; R = Right, L = Left. Whole brain analysis with p < .001 (uncorrected). P-values are FWE-corrected. Anatomical labels were obtained with the anatomical toolbox in SPM12
